# Multiple Strategies for Light-Harvesting, Photoprotection, and Carbon Flow in High Latitude Microbial Mats

**DOI:** 10.3389/fmicb.2018.02881

**Published:** 2018-12-04

**Authors:** Adrien Vigneron, Perrine Cruaud, Vani Mohit, Marie-Josée Martineau, Alexander I. Culley, Connie Lovejoy, Warwick F. Vincent

**Affiliations:** ^1^Centre d’Études Nordiques, Takuvik Joint International Laboratory, Université Laval, Québec, QC, Canada; ^2^Département de Biologie, Université Laval, Québec, QC, Canada; ^3^Institut de Biologie Intégrative et des Systèmes, Université Laval, Québec, QC, Canada; ^4^Département de Biochimie, de Microbiologie et de Bio-informatique, Université Laval, Québec, QC, Canada

**Keywords:** Arctic, microbiome, cyanobacteria, rhodopsin, pigment, biofilm

## Abstract

Microbial mats are ubiquitous in polar freshwater ecosystems and sustain high concentrations of biomass despite the extreme seasonal variations in light and temperature. Here we aimed to resolve genomic adaptations for light-harvesting, bright-light protection, and carbon flow in mats that undergo seasonal freeze-up. To bracket a range of communities in shallow water habitats, we sampled cyanobacterial mats in the thawed littoral zone of two lakes situated at the northern and southern limits of the Canadian Arctic permafrost zone. We applied a multiphasic approach using pigment profiles from high performance liquid chromatography, Illumina MiSeq sequencing of the 16S and 18S rRNA genes, and metagenomic analysis. The mats shared a taxonomic and functional core microbiome, dominated by oxygenic cyanobacteria with light-harvesting and photoprotective pigments, bacteria with bacteriochlorophyll, and bacteria with light-driven Type I rhodopsins. Organisms able to use light for energy related processes represented up to 85% of the total microbial community, with 15–30% attributable to cyanobacteria and 55–70% attributable to other bacteria. The proportion of genes involved in anaplerotic CO_2_ fixation was greater than for genes associated with oxygenic photosynthesis. Diverse heterotrophic bacteria, eukaryotes (including metazoans and fungi) and viruses co-occurred in both communities. The results indicate a broad range of strategies for capturing sunlight and CO_2_, and for the subsequent flow of energy and carbon in these complex, light-driven microbial ecosystems.

## Introduction

Cyanobacterial mats are found in many temperate environments, but are often restricted to hypersaline or hydrothermal environments (Allewalt et al., 2006; Ley et al., 2006) where environmental conditions limit grazer diversity and activity (Stanish et al., 2013; Stal et al., 2017). However, mats are also widespread in alpine, polar and subpolar regions, where they occur in lakes, ponds, and streams. At high latitudes, microbial mats must contend with the extreme conditions of the polar environment (McKnight et al., 2004; Stanish et al., 2013). Comparative analyses of microbial mat communities from high latitudes with those from other regions suggests selection for microorganisms with metabolic pathways that enable survival under conditions such as prolonged freezing, winter darkness and continuous bright light exposure in summer (White et al., 2015; Kleinteich et al., 2017).

Polar microbial mats were initially thought to be relatively simple associations of filamentous cyanobacteria embedded within a mucopolysaccharide gel (Vincent, 1988). However, microscopic and molecular surveys have shown that although structured by filamentous cyanobacteria (de los Ríos et al., 2015), a diverse panoply of microorganisms is found within the mat matrix and interstitial spaces. Co-occurring taxa include many bacterial lineages, autotrophic and heterotrophic protists, fungi, metazoans, and viruses (Vincent et al., 2009; Jungblut et al., 2012; Peeters et al., 2012), suggesting that the mats are complex microbial ecosystems and should be considered as functional consortia. In particular, amplicon and metagenomic surveys have reported a large contribution of proteobacterial lineages within polar mat communities (Bottos et al., 2008; Varin et al., 2010; Lionard et al., 2012; Mohit et al., 2017).

Microbial mats that proliferate at the bottom of shallow lakes and ponds are found from the treeline, across the tundra, to extreme polar desert landscapes (Rautio et al., 2011). Over summer, the maximum day length in the low Arctic (subarctic) to high Arctic regions ranges from 18 to 24 h depending on the latitude, exposing microbial mats to variable conditions of light for photosynthesis as well as UV radiation. Exposure to UV-A and UV-B induce pigment responses and even structural changes in mats; for example, some cyanobacteria migrate away from the surface layers during periods of bright sunlight (Bebout and Garcia-Pichel, 1995; Castenholz and Garcia-Pichel, 2012), while many produce UV-screening and oxidant-quenching pigments (scytonemin, carotenes, and xanthophylls), which benefit all members of the consortia by reducing UV-penetration and the persistence of damaging oxidants in the mats (Quesada et al., 1999).

Although there have been previous metagenomic studies on ice shelf mats exposed to frequent desiccation events (Varin et al., 2010, 2012), the community composition and resulting metabolic potential of mat consortia living in northern rock basin lakes are poorly known. Here we sampled microbial communities located from two sites at opposite ends of the latitudinal range of northern polar microbial mats. At both sites, the mats remain hydrated throughout summer but are frozen over winter. A key difference in habitat is the annual exposure to continuous light and a shorter thaw season in the more northern site. We addressed the hypothesis that mat microbiomes share core taxonomic and functional elements, but that differences in the seasonal light regimes at the two latitudes select for specific pathways for energy acquisition, and for protection against the deleterious effects of sunlight exposure. Our analyses revealed that the mats shared multiple pathways for light-harvesting and carbon flow, with certain differences between locations that may reflect their ambient light conditions.

## Materials and Methods

### Study Sites and Sample Collection

**FIGURE 1:**
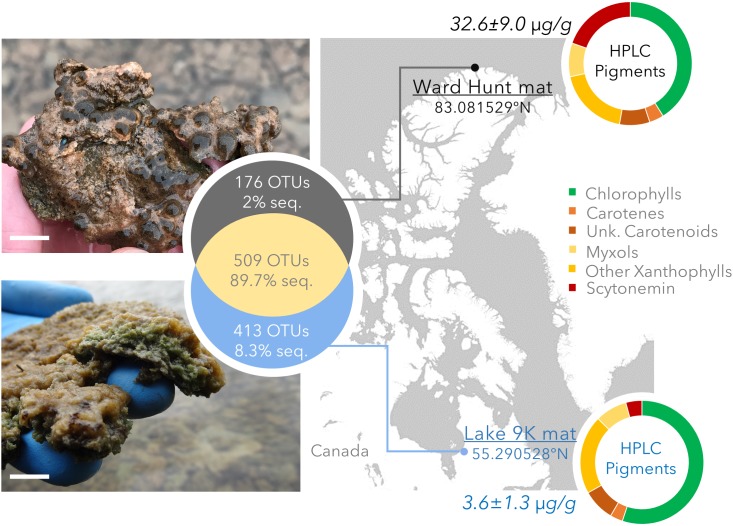
Study sites with geographical position, photographs of the mats, pigment concentration (μg pigment/dry weight mat) and pigment composition determined by HPLC. The top image and pigment profile are for the Ward Hunt Lake mats, and the bottom image and pigment profile are for the Lake 9K mats. Chlorophylls: includes bacteriochlorophylls. Unk.: unknown and non-characterized pigments. The white scale bar represents 1 cm at the center of the photographs. The Venn diagram shows the number of unique and shared OTUs detected by 16S rRNA gene sequencing, with the corresponding percentage of sequences in the whole dataset.

Littoral microbial mats were collected in summer 2015 from Ward Hunt Lake (WHL: latitude 83.081529°N, longitude 74.153330°W) on Ward Hunt Island, off the northern coast of Ellesmere Island (Nunavut, Canada), and Lake 9K (L9K; latitude 55.290528°N, longitude 77.719922°W), near the hamlet of Kuujjuarapik-Whapmagoostui (Nunavik, Canada) (Figure 1). These lie at the northern limit of the high Arctic and the southern limit of the low Arctic, respectively. Both lakes are formed within rocky basins, with little sediment input and with transparent oligotrophic waters. The mats from Ward Hunt Lake had an approximate thickness of 6 mm whereas those from Lake 9K were approximatively 10 mm thick. There are essentially two seasons in the Arctic for these shallow water systems, one frozen (winter) and one thawed (summer), with an abrupt transition between them. WHL microbial mats are exposed to 24 h sunlight in summer with a UV index (noon maximum values^1^) up to 2.5 and water temperatures up to 10°C (4.8°C at the sampling time), whereas L9K region has a maximum of 18 h of daylight in summer with a UV index up to 6 and water temperatures up to 15°C (10°C at the time of sampling). WHL experiences continuous darkness during winter whereas Lake 9K has 18 h of darkness per day in mid-winter, and the seasonal duration of low light is compounded by snow cover for both lakes. Winter temperatures are sufficiently low that both mats are deeply frozen for several months. In the winter of 2014/2015 prior to sampling, daily average air temperatures dropped to around -45°C at Ward Hunt Island (WHL; CEN, 2018) and -38°C at Kuujjuarapik-Whapmagoostui (L9K; CEN, 2017). The depth of freezing is currently around 2 m in WHL and >1 m in L9K.

We collected eight replicate samples at each lake. The samples were collected 3–5 m apart along a transect horizontal to the lake edge, where the water depth was 20–30 cm. The mats were carefully peeled off the rocky substrate and the entire mat sample was immediately placed in RNALater™ (Invitrogen, Carlsbad, CA, United States). After fixing, the samples were maintained at -20°C in the field laboratory and then stored at -80°C until subsequent nucleic acid extraction. Samples for total pigments from two microbial mat subsamples per lake were collected at the same time. These were stored at -80°C, extracted and analyzed by high pressure liquid chromatography (HPLC) as previously described (Jungblut et al., 2009).

### Nucleic Acid Extraction and Preparation

For each replicate sample from Ward Hunt Lake (*n* = 8) and Lake 9K (*n* = 8), nucleic acids were extracted from approximatively 1 g of material. Prior to DNA extraction, each replicate sample was rinsed two times with autoclaved phosphate buffer saline solution to remove the RNALater™. DNA was then extracted from the microbial mats by freeze-thaw lysis (five cycles of freeze-thawing in a high-salinity extraction buffer) followed by chloroform:isoamyl alcohol DNA purification as described (Zhou et al., 1996). The final pellet was re-suspended in 200 μl of DNA-ase and RNA-ase free water, and then purified using a Wizard DNA clean-up kit (Promega, Madison, WI, United States). The purified DNA extracts were stored at -20°C until further analysis.

### Illumina MiSeq Amplicon Library Preparation, Sequencing, and Analysis

The community composition of the microbial mats was determined by sequencing the V4–V5 region of the bacterial 16S rRNA genes (460 bp product) using primers S-D-Bact-0341-b-S-17/S-D-Bact-0785-a-A-20 (Klindworth et al., 2013). The V4 region of the eukaryotic 18S rRNA gene (437 bp product) was amplified using primers E572F/E1009R (Comeau et al., 2011). Attempts to amplify the V1–V3 region of archaeal 16S rRNA genes using primers A27F/Arc518R (Teske and Sorensen, 2007) were not successful in any of the samples. All PCR reactions were conducted in duplicate with negative controls using GoTaq (Promega), with 1 μM of each primer and 1 μl of DNA template in a 25 μl reaction. All PCR assays comprised 30 cycles of denaturation at 95°C for 30 s, annealing for 30 s at the appropriate temperature (58°C for 16S rRNA genes, 55°C for 18S rRNA genes) and extension for 60 s at 72°C followed by a final extension step at 72°C for 7 min. The replicate reaction amplicons were pooled and purified from agarose gels using a Qiagen MinElute Gel purification kit (Qiagen, Hilden, Germany). Indexed amplicons were quantified using a Qubit dsDNA HS Assay Kit (Life Technology, Carlsbad, CA, United States) and diluted to give an equimolar mix of products at a final concentration of 4 nM for MiSeq library preparation. Pair-end Illumina MiSeq sequencing was performed using an Illumina MiSeq v3 kit (Illumina Inc., San Diego, CA, United States), at the IBIS/Laval University, Plate-forme d’Analyses Génomiques (Québec, QC, Canada). Reads were assembled into single pair-end sequences and curated^2^. Overlapping paired-end reads were reassembled using FLASH v2.2.00 (Magoč and Salzberg, 2011) with default settings and extended maximum overlap length (300). CUTADAPT v1.12 (Martin, 2011) with default settings was used to sort paired-end reads by gene region and to remove primers. Sorted sequences were dereplicated, clustered into OTUs (97% similarity) and putative chimeric sequences and singletons were removed using VSEARCH v.2.3.4 (Rognes et al., 2016). Taxonomic affiliations of the reads were determined with mothur (Schloss et al., 2009) using BLAST against Silva database release 128 (Pruesse et al., 2007) as a reference.

### Metagenomic Library Preparation, Sequencing, and Analysis

Metagenomes for six microbial mat samples (three for each lake) were constructed from 1 ng of genomic DNA using a Nextera XT Library Kit (Illumina, San Diego, CA, United States) according to the manufacturer’s recommendations. Tagmentation and indexing were checked using a High Sensitivity DNA chip on an Agilent Bioanalyzer 2100 (Agilent Technologies, Santa Clara, CA, United States). Metagenomes were diluted to 4 nM based on the average DNA fragment size and concentration determined using an Agilent Bioanalyzer 2100 Instrument. The metagenomes were pooled in equimolar quantities and sequenced together using an Illumina MiSeq V3 kit (Illumina, San Diego, CA, United States). Datasets were quality filtered using the Trimmomatic tool (Bolger et al., 2014), with the default setting for paired-end Illumina data to remove residual adaptors and phiX. Pair-end joining was done using the FLASH2 with default settings. Assembly was performed for each sample from paired-end joined reads and unpaired reads passing quality filtering using IDBA_UD (Peng et al., 2012). Assembled contigs and mapping files (BAM file) were uploaded to the IMG/M analysis pipeline (Markowitz et al., 2008) for gene calling and functional annotation. Metagenomes were normalized by multiple (100) rarefactions for sample comparison. Functional annotation was performed against the KEGG database using IMG default settings. The relative abundance of genes with predicted KEGG Orthology was analyzed. For Type I rhodopsin genes (for terminology, see Pinhassi et al., 2016) that were not characterized in the KEGG database, the relative abundance of genes was obtained by BLAST of the reads against public and validated sequences of the gene downloaded from Uniprot. A BLAST hit was considered positive when the bit score was above 50 and the *e*-value greater than e^-15^. Statistical analysis (NPMANOVA, Mann–Whitney *U*-tests) was done using PAST software (Hammer et al., 2001). Raw amplicon sequences were deposited in the NCBI public database under Bioproject PRJNA436954. Metagenome data are available at JGI IMG/M under the following accession numbers: 3300014967, 3300015216, 3300015240, 3300015213, 3300015215, and 3300014966. Details about metagenomes assembly and gene calling are available in Supplementary Table S1.

## Results

### Community Composition

The microbial mat community composition from high Arctic Ward Hunt Lake and low Arctic Lake 9K were determined by both rRNA gene amplicon sequencing (eight replicates per lake) and metagenomic analysis (three replicate samples from each lake). The vast majority (96.6% overall) of metagenomic ribosomal genes (rRNA genes) from all samples were affiliated with *Bacteria*, with *Eukaryota* accounting for 4.4 ± 0.6% of the rRNA reads. *Archaea* were detected in the Lake 9K metagenomes in low proportions (uncultured *Methanomicrobia*, <0.07% of the rRNA reads), but not at all in the Ward Hunt Lake metagenomes. We were not able to amplify archaeal 16S rRNA genes using the archaeal-specific primers Arc787f/Arc1059r.

**FIGURE 2:**
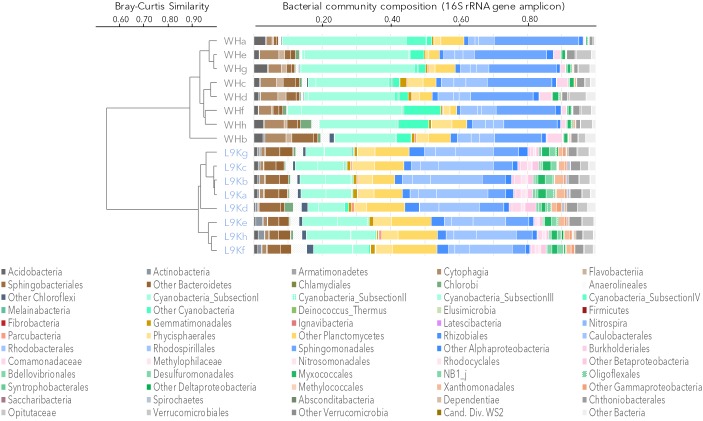
Bacterial community composition based on 16S rRNA gene amplicons. Samples were clustered using the Bray-Curtis similarity measure: Ward Hunt Lake mats (WH), Lake 9K mats (L9K). Major lineages are color coded: Bacteroidetes (browns), Cyanobacteria (light greens), Planctomycetes (yellow), Alphaproteobacteria (blue), Betaproteobacteria (pink), Deltaproteobacteria (dark green), Gammaproteobacteria (orange), and Verrucomicrobia (gray).

The bacterial community composition from 16S rRNA gene amplicons and 16S rRNA gene sequences recovered from the metagenomes was similar in all samples (Pearson correlation coefficient *r* > 0.70, *p* < 0.001). Based on amplicon sequencing, up to 46% (509 OTUs) of the OTUs were shared between WHL and L9K mats. Read counts of the shared OTUs between WHL and L9K mats represented 89.7% of the total reads, indicating that major lineages were shared between the high and low Arctic mats. Non-shared OTUs accounted for 8% of the total reads in L9K mats and 2% of the reads for WHL mats (Figure 1). Despite this strong similarity in dominant taxa, the two bacterial communities clustered apart at the 97% sequence similarity OTU level (Bray-Curtis Similarity index: 55.87; Figure 2) and the community compositions were statistically different (NPMANOVA, *n* = 16, *F* = 27.33, *p* = 0.0002). The difference in ribosomal OTUs was not significant in the metagenomic dataset, where only three samples from each lake were analyzed.

Overall, amplicon sequencing showed that the mat bacterial communities in both the high and low Arctic mats were dominated by members of the Bacteroidetes, Cyanobacteria, Planctomycetes, and Alphaproteobacteria (Figure 2). Additionally, members of Acidobacteria, Chloroflexi, Beta-, Delta-, and Gammaproteobacteria as well as Verrucomicrobia were also identified in both lakes. It is noteworthy that most of the Deltaproteobacteria identified in microbial mats were mainly related to bacterivore non-sulfate reducing lineages of Bdellovibrionales, NB1j, and Myxococcales. There were differences between the two lakes, with Proteobacteria and Chloroflexi having a significantly higher proportion of reads in the L9K compared to WHL mats (Supplementary Figure S1).

Eukaryotic ribosomal sequences in the metagenomes corresponded to 4.4 ± 0.6% of the total reads. The 18S rRNA gene amplicon sequencing indicated a significant difference in eukaryotic community composition between the two lakes (NPMANOVA, *F* = 9.3, *p* = 0.0003). Taxa detected in more than 6 replicate samples per habitat (out of a total of 8) were considered characteristic of the microbial mat from their respective lakes. Five broadly defined taxa corresponded to this criterion in L9K mat (Supplementary Figure S2): the metazoan phyla Nematoda and Rotifera, which are potential bacterivores; Bacillariophyceae (especially pennate diatoms); and two lineages of Cryptomycota, which are potential parasites of diatoms (Gleason et al., 2012). By contrast, 16 eukaryotic taxa were detected in the WHL mat samples (Supplementary Figure S2), including members of the Bacillariophyceae and the green algal order Chaetophorales, heterotrophic Pezizomycotina (fungi in the *Ascomycetes*), metazoa (Nematoda, Rotifera, and Platyhelminthes), ciliates and Cercozoa (heterotrophic protists).

The viral community composition was explored through the taxonomic affiliation of the viral metagenomic reads (0.017 ± 0.001% of total reads). The number of viral sequences and community composition were similar in both mats (*t*-test and NPMANOVA *p* > 0.1) with a predominance of Caudovirales, which are tailed bacteriophages (Myoviridae, Podoviridae, and Siphoviridae) that represented up to 80% of the viral reads (Supplementary Figure S3). Based on closest similarity with known viruses, the main potential hosts of the phages would be *Proteobacteria* and *Cyanobacteria*, which is consistent with the predominance of these two phyla in the bacterial mats (Figure 2).

### Metabolic Potential

The metabolic potential of the microbial communities was evaluated from the metagenomes, with an aim to discover the potential functional traits of the mats. A total of 11,423 different functional genes identified from KEGG orthologies (KO) were detected, with 81% of the KOs occurring in both high Arctic and low Arctic mats (Supplementary Figure S4). These shared genes represented up to 99.92% of the metagenomic reads that were identified. No significant differences between mats from the two lakes were detected in the gene counts for these metabolic potentials (NPMANOVA *p* = 0.1).

**FIGURE 3:**
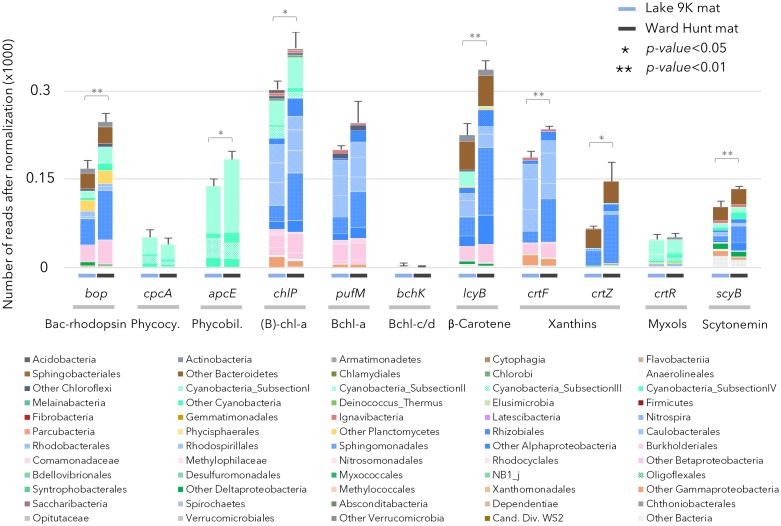
Relative abundance of genes after normalization involved in pigment production in the mat metagenomes for Lake 9K (left bar in each pair) and Ward Hunt Lake (right bar in each pair). The number of asterisks indicates the significance of Mann–Whitney *U*-tests between mean values from Lake 9K and Ward Hunt Lake metagenomes (*n* = 6). Color code of the bars is the same as in Figure 1: Bacteroidetes (browns), Cyanobacteria (light greens), Planctomycetes (yellow), Alphaproteobacteria (blue), Betaproteobacteria (pink), Deltaproteobacteria (dark green), Gammaproteobacteria (orange), and Verrucomicrobia (gray). Bacteriorhodopsin-like protein (Bac-rhodopsin, PFAM family PF01036), Phycocyanin (Phycocy.), Phycobiliprotein (Phycobil.), Chlorophyll-*a* + Bacteriochlorophyll-*a* [(B)-chl-*a*], Bacteriochlorophyll-*a* (Bchl-*a*), Bacteriochlorophyll-*c*/*d* (Bchl-*c*/*d*).

The capacity to capture sunlight by chlorophylls was evident from the abundant presence of genes involved in chlorophyll and bacteriochlorophyll synthesis (*chlP*, Figure 3). Chlorophyll and bacteriochlorophyll share the same initial biosynthetic steps (Heyes et al., 2009) and it is not possible to isolate a gene that is exclusive to chlorophyll synthesis. However, the genes can be separated by phylogenetic analysis, and we found that 25% of the *chlP* sequences were related to Cyanobacteria, indicating the importance of oxygenic photosynthesis by this phylum. Genes for the synthesis of phycocyanin (*cpcA*) and phycobiliproteins (*apcE*) for the phycobilisome complex in cyanobacteria were also detected, as expected. The genes *pufM* and *chlP* involved in bacteriochlorophyll synthesis occurred in both the high Arctic and subarctic microbial mats (Figure 3), indicating the additional importance of aerobic anoxygenic phototrophy. Bacteriochlorophyll-*a* (*Bchl-a*) genes were mainly affiliated to Chloroflexi and aerobic anoxygenic phototrophic bacteria related to Gemmatimonadetes and Alpha-, Beta-, and Gammaproteobacteria. Genes coding for microbial rhodopsins (Type I rhodopsin and bacteriorhodopsin-like proteins; *bop* in Figure 3) were also detected and affiliated to Actinobacteria, Acidobacteria, Bacteroidetes, Cyanobacteria, Planctomycetes, and Alpha- and Betaproteobacteria. Based on the taxonomic affiliation of all of these genes, organisms able to use light for energy-related processes represented up to 85% of the total microbial community, with 15–30% attributable to Cyanobacteria and 55–70% attributable to other bacteria. We also searched for a recently described new class of rhodopsins (heliorhodopsins; Pushkarev et al., 2018). A few reads matched the gene sequences in the heliorhodopsin database: approximately 10 reads for Ward Hunt mats and 30 reads for L9K mats. These were mainly affiliated to Chloroflexi and Actinobacteria.

There were differences between the two sites in the number of genes involved in the production of pigments, with chlorophyll, β-carotene, xanthins, and scytonemin genes significantly more abundant in WHL mats compared to Lake 9K (Figure 3). Extracted pigments identified using HPLC included chlorophylls, carotenes, xanthophylls (including myxols), and scytonemin pigments (Figure 1), covering a range of light-harvesting and photoprotection strategies. Consistent with the number of genes detected, total pigment concentrations per unit dry weight were up to 9 times greater in the Ward Hunt mats (33 ± 9 μg/g) compared to Lake 9K (3.6 ± 1.3 μg/g), with a higher proportion of scytonemin pigments in the Ward Hunt mats (Figure 1).

**FIGURE 4:**
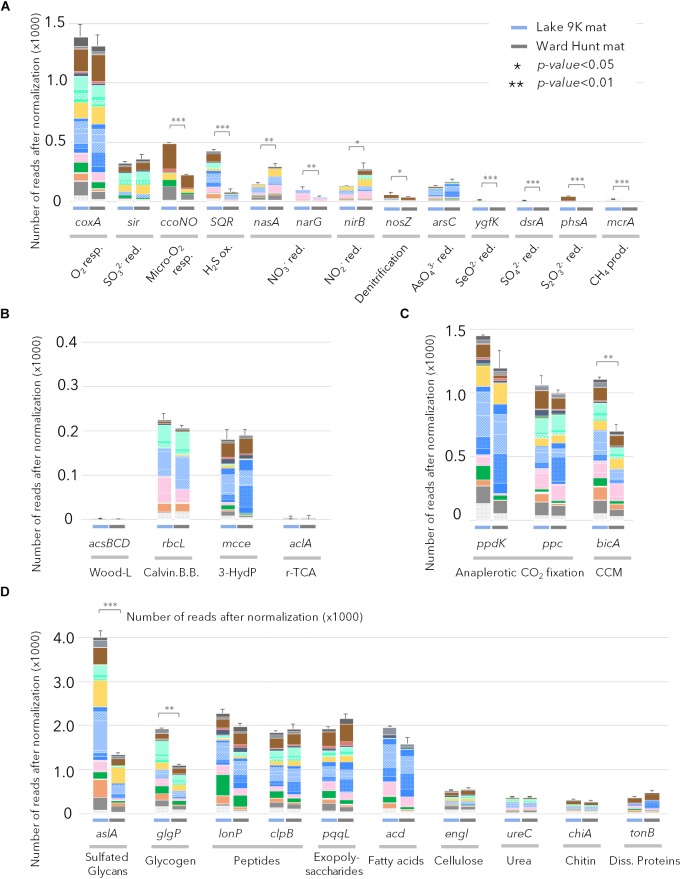
Relative abundance of genes after normalization (see text) involved in **(A)** chemotrophic pathways; **(B)** autotrophic carbon fixation pathways (Wood-Ljungdhal pathway, Calvin-Benson-Bassham cycle, 3-Hydroxypropionate cycle and reverse citrate cycle); **(C)** anaplerotic carbon fixation pathways and CO_2_ concentration mechanisms; **(D)** genes involved in complex carbon degradation detected in Lake 9K metagenomes (left bar in each pair) and Ward Hunt Lake metagenomes (right bar in each pair). Number of asterisks indicates significance of Mann–Whitney *U*-test between mean values from Lake 9K and Ward Hunt metagenomes (*n* = 6). Resp.: respiration; red.: reduction; ox.: oxidation; prod.: production. Color code of the bars is the same as in Figure 1 with Bacteroidetes (browns), Cyanobacteria (light greens), Planctomycetes (yellow), Alphaproteobacteria (blue), Betaproteobacteria (pink), Deltaproteobacteria (dark green), Gammaproteobacteria (orange), and Verrucomicrobia (gray).

The gene profiles showed that energy metabolism was primarily oxygenic in both mats. For example, there were relatively high proportions of sequences coding for the cytochrome c oxidase gene (*coxA*) in the samples from both sites (Figure 4A), and the *coxA* taxonomic affiliations indicated that most members of the microbial community were aerobic. CcoN0 genes, coding for cytochrome cbb3 that is involved in oxygenic respiration under micro-oxic conditions, were detected in larger proportion in L9K compared to WHL mats. These genes were mainly affiliated to groups lacking bacteriochlorophyll: Bacteroidetes, Planctomycetes, Deltaproteobacteria, and Verrucomicrobia. Additionally, genes involved in anaerobic metabolism (nitrate, nitrite, arsenate, selenite, sulfate, thiosulfate reduction, and methanogenesis) were detected, but in low proportions in both lakes. However, these pathways had significantly greater representation in L9K mats compared to WHL mats (Figure 4A).

In terms of inorganic carbon capture, again multiple pathways were evident. The potential for CO_2_ fixation through the Calvin-Benson-Bassham cycle was indicated by the gene *rbcL* encoding for the large subunit of ribulose-1,5-bisphosphate carboxylase/oxygenase (RuBisCO); these sequences were affiliated to Cyanobacteria as well as Rhizobiales and Rhodospirillales (Alphaproteobacteria), Betaproteobacteria, and Gammaproteobacteria. Additionally, CO_2_ fixation via the 3-hydroxypropionate cycle was indicated by methylmalonyl-CoA epimerase genes (*mcce*), and these sequences were affiliated to Bacteroidetes and other Alphaproteobacteria (Sphingomonadales, Caulobacterales, and Rhodobacterales). Finally, there were abundant genes in both mats involved in anaplerotic CO_2_ fixation (PEP carboxykinase – *ppdK* and PEP carboxylase – *ppc*) and in CO_2_ concentration mechanisms (CCM/*bicA*); collectively, up to six times more reads per metagenome were assigned to these C-uptake genes than for the Calvin-Benson-Bassham and 3-hydroxypropionate cycles (Figure 4C), and they were affiliated to almost all phototrophic lineages present in the mats.

Many catabolic pathways were identified in both habitats (Figure 4D). These included the metabolic potential for degradation of various complex carbon substrates, including glycans (*aslA*), glycogen (*glgP*), polysaccharides (*pqqL*), and fatty acids (*acd*). The number of genes involved in recycling microbial mat metabolic products (extracellular matrix, denatured proteins, carbon storage, secondary metabolites) far exceeded the most represented genes for degradation of particles that could also originate from outside the mats (cellulose, chitin, urea, and dissolved proteins). Based on taxonomic affiliation of the catabolic genes, Proteobacteria, Bacteroidetes, Planctomycetes, and Verrucomicrobia were the primary decomposers of proteins, peptides, and fatty acids (Figure 4D). The potential to degrade intracellular damaged polypeptides and recycle amino acids (*Lon* and *Clp* proteases) was also detected in the microbial mat communities, along with the capacity for carbon storage (glycogen) and degradation.

## Discussion

### Core Microbiome Features

Mats from both the high and low Arctic sites were complex microbial systems, with multiple phylogenetic lineages including Cyanobacteria, Bacteroidetes, Planctomycetes, Alpha-, Beta-, and Gammaproteobacteria, and microbial and metazoan eukaryotes (Figure 2 and Supplementary Figure S2). There was considerable overlap in taxonomic composition and functional capacities in the two Arctic microbial communities (Figures 1–3 and Supplementary Figure S4), suggesting a taxonomic and metabolic core microbiome consistent with active carbon cycling in polar mats (Singh and Elster, 2007; Jungblut et al., 2017). Taxonomic and functional profiles were similar to subarctic microbialites, that harbor abundant members of the Alphaproteobacteria with phototrophic capabilities (White et al., 2015). Many of the same taxa were also previously reported in metagenomic studies on mats collected from Arctic and Antarctic ice shelves (Varin et al., 2010, 2012). Ice shelves are thick (tens to hundreds of m) layers of ice floating on the sea and are often highly oligotrophic systems; the ice shelf mats found in surface melt ponds may be subjected to frequent desiccation following winter freezing and release from the ice (Varin et al., 2010). In contrast the lake mats investigated here are subject to less extreme conditions, and more likely to inhabit the same physical space over multiple years, which would suggest that the taxonomic functional characteristics represent adaptation to the *in situ* conditions where they were collected.

Cyanobacteria along with associated diatoms and certain other eukaryotic algae have long been thought to be the main agents of primary production in polar microbial mats, via oxygenic photosynthesis (Vincent, 2002). However, our metagenomic analysis revealed that bacteriochlorophyll (*pufM*) and Bac-rhodopsin (*bop*) genes exceeded the number of chlorophyll-*a* synthesis genes, indicating that bacteriochlorophyll and Bac-rhodopsin light-harvesting processes are also likely to be quantitatively important (Figure 3). Bacteriochlorophyll genes have been previously detected in other cyanobacterial mats and stromatolites (Ranchou-Peyruse et al., 2006; Stal et al., 2017). The taxonomic affiliation of bacteriochlorophyll and Type I rhodopsin genes in the metagenomic dataset suggest that up to 70% of the bacterial community including members of the Actinobacteria, Acidobacteria, Bacteroidetes, Planctomycetes, Gemmatimonadales, Alpha-, Beta-, and Gammaproteobacteria had the metabolic potential for some form of phototrophy. In total, phototrophic microbes of diverse lineages and light-harvesting pathways represented up to 85% of the microbial mat community. It has been recently proposed that bacteriochlorophyll and proteorhodopsins, which are also Type I rhodopsins, potentially absorb as much as or more light energy than chlorophyll and that their cellular energy yield may be sufficient to sustain bacterial basal metabolism (Gómez-Consarnau et al., 2017). In addition, rhodopsin genes occur in microbial eukaryotes, such as the eukaryotic clade of proteorhodopsin detected in Arctic fjord plankton (Vader et al., 2017). The coexistence of multiple light-capturing mechanisms is consistent with different microbes selectively able to use energy in different parts of the light spectrum, creating opportunities for niche differentiation (Stomp et al., 2007; Larkum et al., 2018).

The metagenomic survey and pigment data highlighted the widespread occurrence of UV-protecting pigments (scytonemin, carotenes such as β-carotene, xanthins, and myxol compounds; Figure 1). These pigments protect microorganisms from near UV and visible high light damage by sun-screening (scytonemin) or by quenching triplet-state photosensitizers and reactive oxygen species (carotenoids) (Tong and Lighthart, 2008; Maccario et al., 2015). Although it might reflect the difference of thickness between high and low Arctic mats, the concentration of these pigments, as well as number of genes involved in pigment synthesis, was significantly greater in Ward Hunt Lake microbial mats. In this northern lake, the microbial mats face continuous light exposure in summer but a lower UV index due to the lower incidence angle of the light compared to the low Arctic mat. The larger proportion of phototrophic genes and pigments is consistent with the positive correlation between anoxygenic phototroph abundance and day length in freshwater environments (Cepáková et al., 2016; Garcia-Chaves et al., 2016) and suggests that the total duration of light exposure rather than maximum UV irradiance may shape community structure and pigmentation. The metabolic potential for photoprotective pigment synthesis was common to both microbial mat communities, suggesting that most cells in the consortia are protected from at least part of the damage from the long daily exposure to solar radiation (Figure 3).

Cyanobacteria, Proteobacteria, and Bacteroidetes showed a capacity to synthesize pigments covering different wavelengths of the light spectrum. This broad wavelength screening as well as oxidative stress protection is likely to contribute to their ecological success in the microbial mat communities. The metabolic capacity to protect against the deleterious effects of UV exposure would also confer a strong selective advantage in polar environments where solar radiation is continuous, with little or no opportunity for full repair and recovery during night-time darkness (Mueller et al., 2005; Bonilla et al., 2009).

The aerobic anoxygenic phototrophs known to date are photoheterotrophs that have the capacity to obtain energy from both oxidation of organic matter and light-induced proton transport, thereby providing a competitive advantage under light exposure (Garcia-Chaves et al., 2016; Ferrera et al., 2017). Phototrophy has been proposed to confer an additional selective advantage in oligotrophic waters by promoting anaplerotic CO_2_ fixation pathways under light stimulation (Palovaara et al., 2014; Ferrera et al., 2017). Genes coding for anaplerotic CO_2_ fixation and CO_2_ concentration mechanisms were affiliated with organisms also able to produce bacteriochlorophyll and Bac-rhodopsin, and they were up to six times more represented than any other CO_2_ fixation pathways (including the Calvin-Benson-Bassham cycle in Cyanobacteria) in both high and low Arctic microbial mats (Figures 4B,C). This suggests that light-induced anaplerotic CO_2_ fixation represents one of the main carbon pathways in microbial mats, along with photosynthesis by Cyanobacteria, diatoms and other algae. Non-RuBisCO-based carbon metabolism, including anaplerotic CO_2_ fixation, as well as reductive TCA cycle and 3-hydroxypropionate cycle genes that were also identified in our dataset, have been detected in Shark Bay hypersaline microbial mat metagenomes (Ruvindy et al., 2015), and may represent a general feature of microbial mats.

### Functional Model of the Polar Mat Microbiome

**FIGURE 5:**
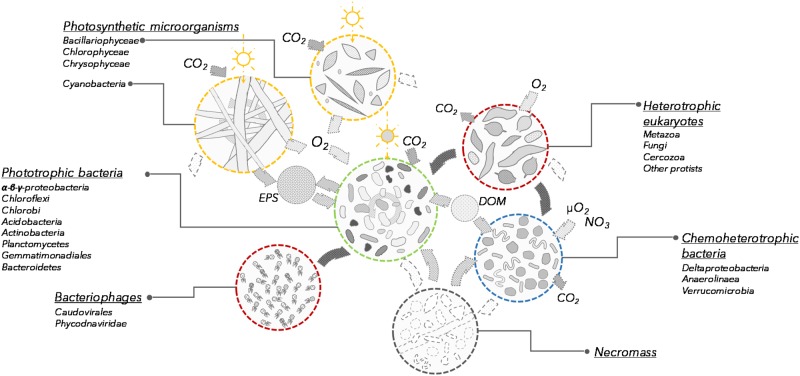
Conceptual model of the communities and processes involved in microbial mat functioning (not to scale). Each group is characterized by its potential function in the microbial mat ecosystem. Open sun indicates photosynthesis and dark sun indicates bacteriochlorophyll and bacteriorhodopsin phototrophy. EPS, exopolysaccharides; DOM, dissolved organic matter.

Based on the taxonomic profiles and the metabolic potential identified in the microbial mat samples, we propose a revised conceptual model for microbial mat functioning where photosynthetic and other pigmented microorganisms co-dominate and are responsible for mat growth and expansion over timescales of years (Figure 5). Multiple strategies for atmospheric CO_2_ fixation were identified, implying that primary production in these microbial systems is distributed across a variety of light-utilizing organisms.

Cyanobacteria produce diverse exopolysaccharides, and these extracellular polymers contribute to the engineering of the microbial mat ecosystem, sheltering eukaryotic and prokaryotic microorganisms in a high-nutrient, secondary metabolite-rich microenvironment (Vincent et al., 2000; Harada, 2004; Bonilla et al., 2005). Numerous genes involved in the degradation and oxidation of exopolymers, glycogen, denatured peptides and fatty acids were detected in our samples and were related to the same bacterial orders as aerobic anoxygenic phototrophs. Although the communities probably also included non-phototrophic bacteria from these orders, our metagenomic analyses indicated that along with the anaplerotic CO_2_ fixation pathway, the phototrophic non-cyanobacteria might have a strong metabolic potential for degradation of exopolymers, glycogen, denatured peptides and fatty acids and organic matter, suggesting that some of these taxa have a mixotrophic lifestyle (Figure 4D). This would be consistent with the known heterotrophic capabilities of aerobic anoxygenic phototrophic bacteria (Garcia-Chaves et al., 2016) as well as bacteria with Bac-rhodopsin (Maresca et al., 2018). This capacity to degrade organic matter and assimilate cyanobacterial and diatom exudates may be a form of mutualism, as the association with bacterial communities is reported to enhance cyanobacterial growth (Berg et al., 2008). In addition, this catabolic potential could also be used to recycle necromass (dead cells and other cellular debris) generated by lethal environmental conditions or potential attacks of phages (Anesio and Bellas, 2011), eukaryotic grazers (Nematoda, Rotifera, ciliates, nanoflagellates) and predatory bacteria such as Bdellovibrio and Myxobacteria (Deltaproteobacteria), as identified here (Supplementary Figures S2, S3). The presence of eukaryotic grazers and a phage community suggest the occurrence of continuous bacterial mortality and nutrient recycling.

Chemoheterotrophic microorganisms (Deltaproteobacteria, Anaerolineae, Planctomycetes, and Verrucomicrobia) were also identified within the microbial mats, as reported elsewhere (Berg et al., 2008; Bruckner et al., 2008). However, the relative proportion of these lineages was low compared to marine microbial or intertidal cyanobacterial mats (Ruvindy et al., 2015). The metabolic potential for degradation of organic exudates, necromass, but also cellulose, urea and other exogenous dissolved proteins was identified in metagenomic reads affiliated to these lineages, supporting previous metagenomic profiling (Varin et al., 2010; Ruvindy et al., 2015). Aerobic respiration and organic carbon degradation appear to be the main energy pathways for the non-photosynthetic microbial community (Figure 4D). Genes affiliated with Bacteroidetes, Deltaproteobacteria, Planctomycetes, and Verrucomicrobia lineages involved in oxygen respiration in microoxic conditions were also identified, suggesting that chemoheterotrophic bacteria would be most likely found in the deepest layers of the microbial mat where oxygen concentrations would be limited (Paerl et al., 2000; Lionard et al., 2012) and contribute to the recycling of carbon and nutrients within the microbial mats (Figure 4A). Anaerobic microbiota such as methanogens, sulfate reducers and anaerobic photosynthetic bacteria were conspicuously absent or poorly represented. This likely reflects the shallow water habitat, which is highly oxygenated in summer, and then freezes solid during winter. This contrasts with consistently unfrozen microbial mats in deeper polar lake environments, where metabolism continues during winter darkness and may drive the environment toward anoxia and anaerobic processes, such as methane generation (Mohit et al., 2017), and also differs from partly anoxic and sulfate-rich marine microbial mats (Ranchou-Peyruse et al., 2006; Ruvindy et al., 2015). Genes involved in chemosynthetic pathways were detected in larger proportion at the southern latitude site (Lake 9K; Figures 1, 4A). This might be favored by the greater thickness of the mat generating anaerobic micro-niches, or may reflect a potential for dark reactions in southern microbial mats, increased oxygen demand due to warmer temperatures, and the influence of allochthonous inputs of plant-derived organic materials from the more vegetated catchment.

The metabolic capacities identified in our study remain to be confirmed by larger scale investigations, including activity measurements and transcriptomic analyses across many northern as well as south polar lakes. However, the results all point to light utilization and protection by multiple mechanisms as a key component of the polar mat microbiome, along with multiple pathways of carbon fixation and flow in these complex, light-driven microbial ecosystems.

## Author Contributions

AV, WV, and CL designed the research. WV, AC, and VM collected the samples. AV, PC, and VM analyzed the data. M-JM and WV contributed the HPLC data. AV, WV, and CL led the writing of the paper, with contributions from AC. All authors commented on the final version of the manuscript.

## Conflict of Interest Statement

The authors declare that the research was conducted in the absence of any commercial or financial relationships that could be construed as a potential conflict of interest.

## Footnotes

1 http://exp-studies.tor.ec.gc.ca/

2 https://github.com/CruaudPe/MiSeq_Multigenique
