# Supplementary material for: Multiple Strategies for Light-Harvesting, Photoprotection, and Carbon Flow in High Latitude Microbial Mats
Source: Front Microbiol. 2018 Dec 4;9:2881. doi: 10.3389/fmicb.2018.02881 (PMC6288179; doi:10.3389/fmicb.2018.02881)
Supplement: Supplementary file 1 [file Data_Sheet_1.docx]

***Supplementary Material***

**Multiple Strategies for Light-Harvesting, Photoprotection and Carbon Flow in High Latitude Microbial Mats**

Adrien Vigneron^1,2,3^, Perrine Cruaud^3,4^, Vani Mohit^1,2,3^, Marie-Josée Martineau^1,2^, Alexander I. Culley^1,3,4^, Connie Lovejoy^1,2,3^ and Warwick F. Vincent^1,2,3^

**Supplementary Figures and Table**

**
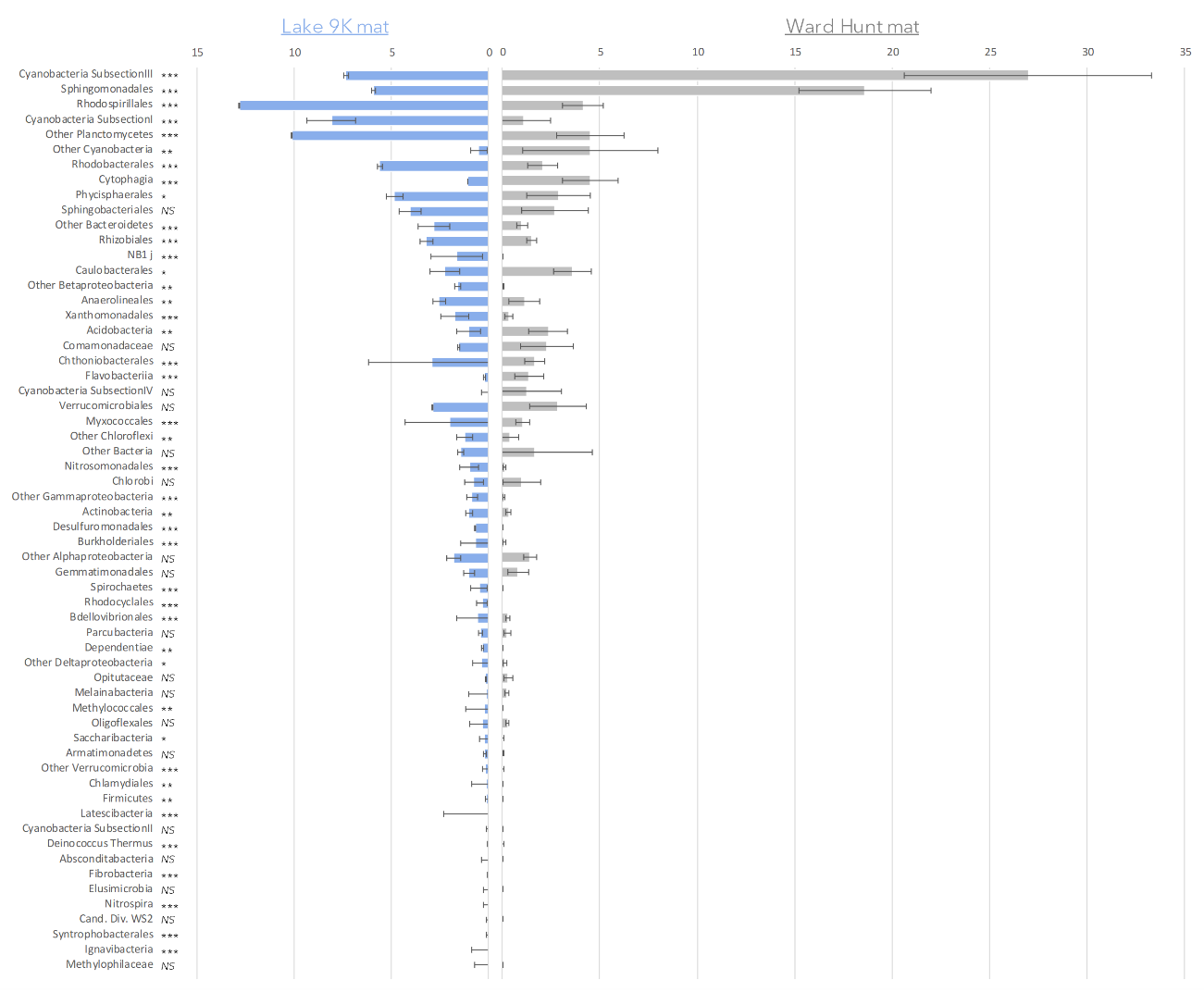
**

**Supplementary Figure S1.** Relative abundance of bacterial lineages in Lake 9K and Ward Hunt mats. Lineages were sorted by descending contribution to the dissimilarity observed between the two habitats (SIMPER data). Number of asterisks indicates significance of Mann-Whitney U test between mean values from Lake 9K and Ward Hunt Lake samples (n=16): ***: p<0.0001 **: p<0.001, *:p<0.01.


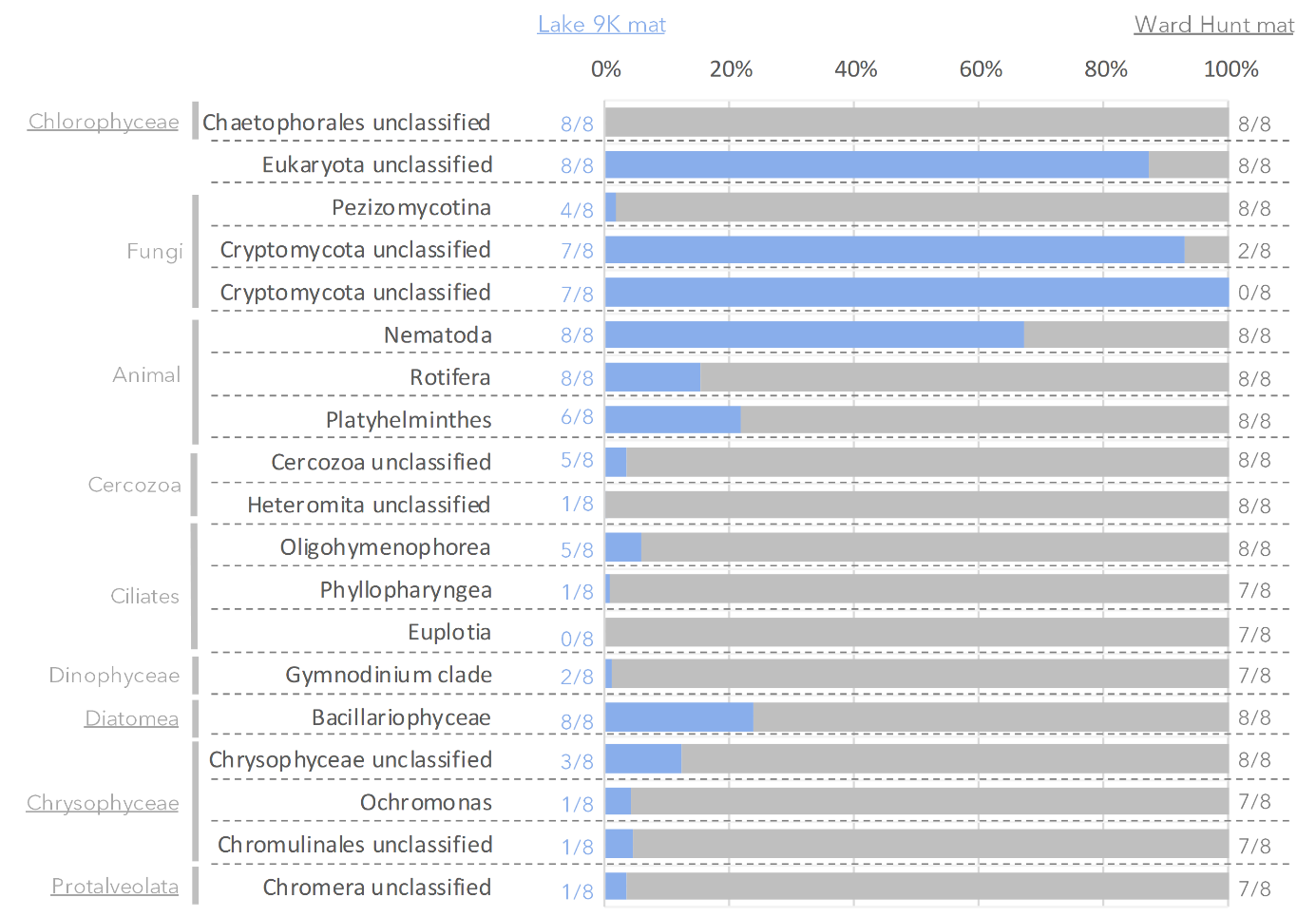


**Supplementary Figure S2.** Relative proportion of Eukaryotic lineages detected by 18S rRNA gene sequencing between Lake 9K and Ward Hunt Lake mats. Only lineages detected in more than 6/8 replicates in at least one habitat were considered as systematic components of the mats and were represented.


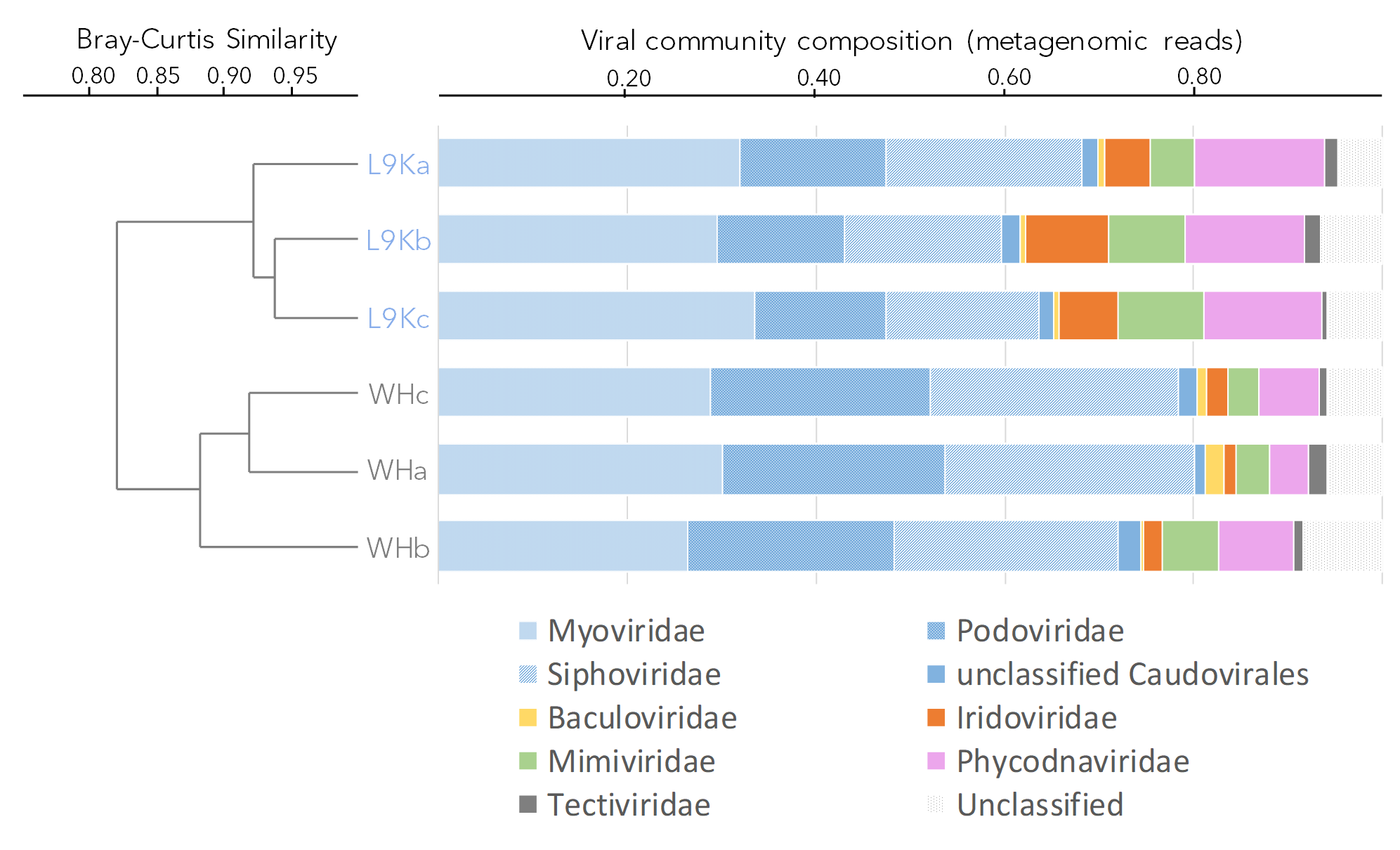


**Supplementary Figure S3.** Viral community composition based on taxonomic affiliation of metagenomic reads. Shades of blue indicate Caudovirales.


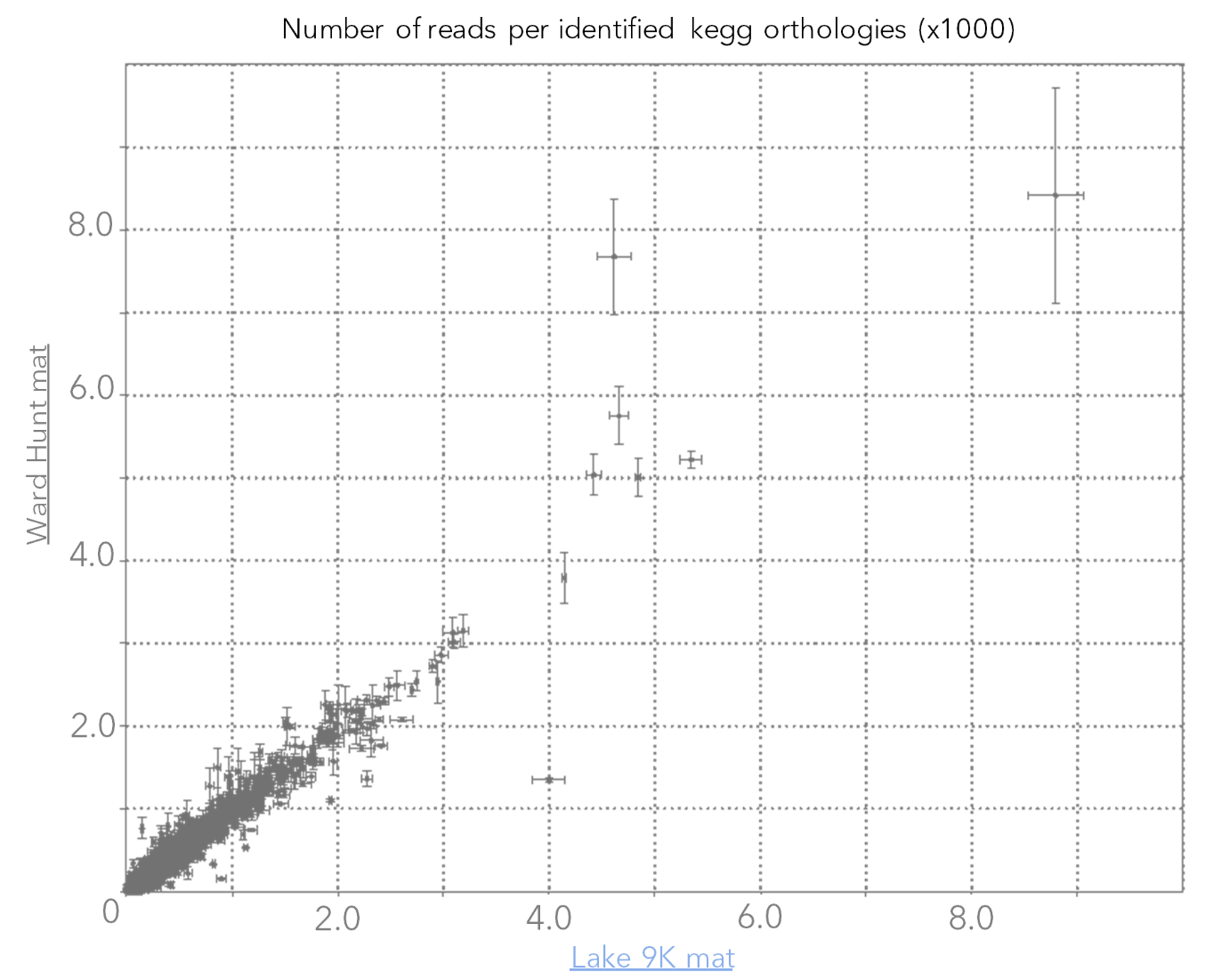


**Supplementary Figure S4.** Number of reads per identified genes (Kegg orthology) in Lake 9K and Ward Hunt Lake metagenomes (n=6). Each dot represents a single KO. Dots follow a 1:1 distribution, indicating that most of the genes were detected in equal proportion in both microbial mat habitats.

**Supplementary Table 1.** Metagenomic sequencing, assembly and gene calling details for the replicate (a,b,c) microbial mat samples. WH: Ward Hunt Lake mat; L9K: Lake 9K mat.

| Sample Name | Genome Size (bp) | Contig Count | Average contig size | N50 | Gene Count | RNA Count | Genes with predicted function | KEGG Count | KO Count | IMG Genome ID |
| --- | --- | --- | --- | --- | --- | --- | --- | --- | --- | --- |
| WHa | 817233659 | 3218407 | 648 | 702 | 3233833 | 9693 | 3233833 | 634761 | 1044833 | 3300014966 |
| WHc | 867187793 | 3598524 | 494 | 469 | 3585381 | 11171 | 3585381 | 732620 | 1191717 | 3300015213 |
| WHb | 912092833 | 3822884 | 544 | 514 | 3797184 | 11986 | 3797184 | 781991 | 1264240 | 3300015215 |
| L9Ka | 933455729 | 4141771 | 489 | 476 | 4110994 | 11935 | 4110994 | 842376 | 1360233 | 3300014967 |
| L9Kb | 1055569398 | 4339506 | 501 | 493 | 4354582 | 12957 | 4354582 | 896225 | 1452913 | 3300015216 |
| L9Kc | 1268020347 | 5259175 | 507 | 493 | 5277835 | 14769 | 5277835 | 1089219 | 1764663 | 3300015240 |
